# Innovative voltammetric techniques for bumadizone analysis in pharmaceutical and biological samples: emphasizing green, white, and blue analytical approaches

**DOI:** 10.1038/s41598-024-69518-w

**Published:** 2024-08-27

**Authors:** Khadiga M. Kelani, Ragab A. Said, Mohammad A. El-Dosoky, Ahmed R. Mohamed

**Affiliations:** 1 Pharmaceutical Analytical Chemistry Department, Faculty of Pharmacy, Cairo University, El-Kasr El-Aini Street, Cairo, 11562 Egypt; 2 Pharmaceutical Analytical Chemistry Department, Faculty of Pharmacy, Al-Azhar University, Nasr City, Cairo, 11751 Egypt; 3 Chemistry Department, Faculty of Pharmacy, Heliopolis University for Sustainable Development, 3 Cairo Belbeis Desert Road, Cairo, Egypt; 4 Pharmaceutical Chemistry Department, Faculty of Pharmacy, Egyptian Russian University, Badr City, Cairo, 11829 Egypt

**Keywords:** Bumadizone, Square wave voltammetry, Nano-reduced graphene-oxide, Carbon paste electrode, Electrochemical impedance spectroscopy, Chronoamperometry, Environmental sciences, Nanoscience and technology

## Abstract

There are no documented electroanalytical methods for quantifying the anti-inflammatory drug bumadizone (BUM) in pharmaceutical or biological matrices. So, a new voltammetric method was developed to determine BUM at nano concentrations in pharmaceutical forms, in the presence of its alkaline degradant, and in biological fluids. Five electrodes were tested, including three nano-reduced graphene oxide (nRGO) electrodes (5%, 15%, and 20%), a carbon paste electrode (CPE), and a 10% nRGO-modified CPE. The 10% nRGO-modified electrode showed the best performance, offering high selectivity and low detection limits, with good linearity in the concentration range of 0.9 × 10^2^ to 15 × 10^2^ ng mL^−1^. Differential pulse voltammetry successfully applied this electrode for BUM determination in various samples, achieving excellent recovery without preliminary separation. The method was validated according to ICH guidelines and compared favorably to the reference method. Its environmental impact was assessed using AGREE and Eco-scale metrics in addition to the RGB algorithm, showing superior greenness and whiteness profiles due to safer solvents and lower energy consumption, along with high practical effectiveness using the BAGI metric.

## Introduction

Bumadizone calcium hemihydrate is calcium 2-(1,2-phenylhydrazine carbonyl) hexanoate hemihydrate^1^. It is a nonsteroidal anti-inflammatory drug (NSAID) with analgesic properties, marketed for the treatment of rheumatoid arthritis, gout, and post-traumatic oedema^2,3^. It acts by blocking prostaglandin biosynthesis by inhibiting cyclooxygenase enzyme (COX). It is metabolized to phenylbutazone and oxyphenbutazone, and its use has been limited by the risk of agranulocytosis and other hematological adverse effects^1^. Few analytical methods are available for the quantitative determination of BUM using spectrophotometric analysis^4–6^, oxidative coupling reaction^5^, spectrofluorometric method^7^, thin layer chromatographic HPTLC densitometry^6^, and high-performance liquid chromatographic (HPLC)^6,8^ methods. Stability-indicating assay methods of the first derivative (1D), ratio spectra (1DD), iso-absorptive point spectrophotometry, and ratio subtraction^9^ were also reported.

Electrochemical methods offer numerous advantages over conventional techniques, including heightened sensitivity, exceptional selectivity, and extensive analytical capabilities, such as accommodating a wide range of concentrations and pH levels. Additionally, they boast affordability, utilize non-toxic chemicals, require minimal analysis time, and are applicable even to turbid or colored solutions. Consequently, the continued advancement and utilization of voltammetric methods remain pivotal in pharmaceutical analysis^10–14^. However, to date, there are no documented electroanalytical methods for quantifying BUM, either in its bulk state, pharmaceutical formulations, or biological fluids or for its simultaneous determination alongside its alkaline-induced degradant, diphenyl hydrazine (DPH), to the best of the authors’ knowledge.

In this study, we introduce a novel electroanalytical approach utilizing square wave voltammetry (SWV), employing nano-reduced graphene oxide (nRGO) and carbon paste (CPE) electrodes. This method enables the precise quantification of the drug at nano-concentration levels, whether in its pure form, pharmaceutical preparations, or in the presence of its alkaline degradant. Remarkably, the technique eliminates the need for cumbersome and time-consuming separation steps, making it particularly convenient for practical applications, including the analysis of spiked serum and urine samples. The developed method offers exceptional sensitivity and selectivity for the determination of BUM, showcasing its potential as a valuable analytical tool. Notably, its utilization of non-hazardous chemicals and the absence of toxic waste make it environmentally safe, aligning with the growing emphasis on greener analytical practices. Greening analytical procedures are of paramount importance and are receiving much attention in order to minimize the negative environmental impacts by substituting non-green analytical methods with more eco-friendly alternatives that consume and generate less toxic solvents^15–17,18^. Furthermore, the method significantly reduces analysis time, enhancing efficiency in laboratory workflows.

## Experimental

### Apparatus

All voltammetric measurements were carried out using a Metrohm computrace voltammetric analyzer model 797 VA with Software Version 1.0 (Metrohm Switzerland) for voltammetric analysis equipped with three-electrodes: an Ag|AgCl (3 M KCl) electrode as the reference electrode, a platinum electrode as an auxiliary electrode, and working electrodes (nRGO and CPE electrodes). The pH measurements were carried out using a digital pH meter, Jenway 3510, with a combined glass electrode.

Scanning electron microscopy (SEM) measurements were carried out using a JSM-6700F scanning electron microscope (Japan Electro Company). All the electrochemical measurements were performed at an ambient temperature of 25 ± 2 °C.

Electrochemical impedance spectroscopic measurement was performed using a Gamry-750 system and a lock-in-amplifier connected to a personal computer. The data analysis software was provided with the instrument, and a non-linear least square fitting was applied.

### Materials and reagents

All solvents and reagents used were of grade (A) purity to avoid the need for additional purification steps.

Pure standard BUM calcium hemihydrate was kindly supplied by October Pharma S.A.E. Company, 6th October City, Egypt. Its purity was found to be 99.98% using the reported method^6^.

Octomotol® tablets (batch no. B4361009) purchased from the local market were labeled to contain 110 mg of BUM; they were manufactured by October Pharma. S.A.E. Company, 6th October City, Egypt.

nRGO, paraffin oil, and graphite powder (particle dimension 20 μm) were purchased from Sigma-Aldrich, Germany.

HCl (1 N and 0.1 N aqueous solutions) and NaOH (1 M and 0.2 M prepared by dissolving 40 gm and 8 gm of NaOH in 1L distilled water) were obtained from Sigma-Aldrich, Germany.

Britton Robinson (BR) buffer 0.04 M, used for the range of pH 2 to pH 12, was prepared by mixing different volumes of 0.04 M phosphoric acid (ADWIC, Egypt), 0.04 M glacial acetic acid (ADWIC, Egypt), and 0.04 M boric acid (ADWIC, Egypt) with an appropriate amount of 0.2 M sodium hydroxide to obtain pH over the range of 2.0–12.0.

Glacial acetic, boric, orthophosphoric acid, 5% ZnSO_4_, ethanol, and methanol were supplied by Merck, Darmstadt, Germany. Sodium dodecyl sulfate (SDS) was the product of Qualikems, India.

### Standard solutions

#### Standard stock solution of BUM

A standard stock solution of BUM (0.8 mg/mL) was freshly prepared by precisely weighing and transferring 20 mg of pure BUM into a 25 mL volumetric flask. Subsequently, it dissolved in 1 mL of methanol, and the volume was completed to the mark with distilled water.

#### Preparation of standard stock solution of BUM alkaline-induced degradant

The alkaline degradant of BUM was prepared by weighing 50 mg of BUM powder in a conical flask and then refluxing with 15 mL of 1 M sodium hydroxide solution for 7 h. Complete degradation was followed via TLC using hexane–ethyl acetate–glacial acetic acid (8:2:0.2 v/v) as a developing system. After complete degradation, the solution was neutralized with 1 M HCl to pH 7. The solution was evaporated under vacuum and the residue was extracted with multiple fractions of ether. The ethereal extract was evaporated at room temperature in a fuming cabinet. The residue was then used for preparation of the stock solution of the alkaline degradation product DPH (claimed to contain 1 mg/mL in methanol) by weighing 25 mg of DPH residue into a 25 mL volumetric flask; 20 mL of methanol was added to the flask and shaken to dissolve, then the volume was made up to the mark with methanol^6^.

### Analytical procedures

#### Preparation of working electrodes

- *Carbon Paste Electrode* (CPE) was prepared by mixing 0.3 g graphite in a mortar with 120 µL paraffin oil to form a homogenous paste. The paste was packed into the hole of the insulin syringe body with a diameter of 3.0 mm, at which a copper wire was placed for connection of the constructed electrode with the apparatus. The tip of the designed electrode was polished using white paper until it showed a shiny appearance^19^.
- *Nano-Reduced Graphene-Oxide Electrodes* (nRGO): three bulk modified electrodes were prepared in percentages of 5, 15, and 20% by weight-appropriate amounts of nRGO (0.015 gm, 0.045 gm, and 0.060 gm), respectively, and completing the weight of electrodes with graphite to 0.3 gm, then adding 150 µL paraffin oil to each to form homogenous pastes. Portions of these pastes were packed separately into the holes of the insulin syringe bodies with a diameter of 3.0 mm, at which a copper wire was connected to the apparatus. A surface 10% nRGO electrode was prepared by mixing 5.0 mg nRGO and 50 mL dimethylformamide and sonicating for 30 min. Then, 20 µL of the solution was added to the tip of the carbon paste electrode and left to evaporate in the open air to form a surface of nRGO-modified electrode, and this was repeated 3 times^19^.

#### Voltammetric behavior of BUM at nRGO and CPE electrodes

The voltammetric behavior of BUM at nRGO and CPE electrodes was recorded through cyclic voltammograms for cyclic and square wave voltammetry in BR buffer over the pH range of 2.0–12.0 using 15 mL of the buffer solution containing 50 µL of the drug added to voltammetric cell compartment. This was followed by immersion of the electrode, stirring the component for 10 s (the proposed accumulation time) using 0.4 V applied potential for the electrode, followed by a 5-s stop to allow the solution to become quiescent. The voltammograms were then recorded at a scan rate of 100 mVs^−1^ with an applied potential of 0.4 to 1.1 V against the Ag|AgCl|KCl reference electrode^20^.

The cyclic wave (CW) and differential pulse (DP) voltammograms of 5 × 10^–5^ M, 1 × 10^–3^ M of BUM, and 1 × 10^–3^ M of its alkaline degradant were recorded under the same optimum experimental conditions with the use of SDS surfactant against a blank of the supporting electrolyte (BR buffer solution). The recorded cyclic voltammograms reflecting the voltammetric behavior of BUM and its degradation products at nRGO and CPE electrodes in BR buffer pH 3.0 exhibited a well-defined and sharp anodic peak at + 0.92 V. Meanwhile, the differential pulse (DP) voltammograms of the same concentrations and conditions of BUM also showed a single peak at + 0.7 V. The differential pulse (DP) voltammograms of the corresponding alkaline degradant were also collected and compared with those obtained from the intact drug at the same previously stated concentrations. All data were obtained at room temperature.

### Optimization of experimental parameters affecting voltammetric responses

Various experimental conditions affecting voltammetric responses were investigated and optimized in this study^20^. These conditions included pH, scan rate, accumulation time and potential, surface area, chronoamperometry, electrochemical impedance, and the use of surfactants. The cyclic voltammograms were recorded using 0.05 × 10^−5^ M of BUM at nRGO and CPE. The effect of pH was investigated across the range of 2.0–12.0 at a scan rate of 100 mVS^−1^. The impact of scan rate and spanning from 20 to 500 mVS^−1^ was examined to assess its influence on both peak current (Ip) and peak potential (Ep). Additionally, the effects of accumulation time (ranging from 0 to 60 s) and accumulation potential (ranging from − 0.6 to 0.1 V) on the peak current were investigated. The surface area of the selected electrode was determined using 1.0 mM K_3_Fe(CN)_6_ while applying serial scan rates. The Randles–Sevcik equation^21^ was applied for calculation.

#### Chronoamperometric measurements

Chronoamperometric measurements of BUM at 10% nRGO electrode were studied by setting the electrode potential at + 850 mV; chronoamperograms were obtained using various BUM concentrations (3.3, 6.6, 10, 13.3, 16.6 × 10^–5^ μmol L^−1^) in BR buffer (pH 3). Chronoamperometric measurements of BUM were conducted using a 10% nRGO electrode, with the electrode potential set at + 850 mV. Chronoamperograms were recorded at various BUM concentrations (3.3, 6.6, 10, 13.3, 16.6 × 10^–5^ μmol L^−1^) in BR buffer (pH 3). These experiments enabled the characterization of the electrochemical behavior of BUM at the selected electrode potential and provided insights into its concentration-dependent response under the given experimental conditions.

#### Electrochemical impedance spectroscopy (EIS) measurements

An oxidation potential with an amplitude of 810 mV was applied to both the nRGO and CP electrodes across a frequency range spanning from 0.1 Hz to 100 kHz. These measurements were conducted in a solution containing 10 mmol L^−1^ of BUM in BR buffer (pH 3). Subsequently, a typical impedance spectrum was obtained, allowing for the characterization of the electrochemical impedance behavior of the system.

### Construction of analytical curve

Precise aliquots of BUM standard stock solution (0.025–1.0 mMol) were carefully transferred into 25-mL volumetric flasks, and the solution in each flask was completed to the mark with 0.04 M BR buffer (pH 3.0). Utilizing an nRGO electrode, the square wave (SW) and differential pulse (DP), voltammograms were recorded in the range of 0.4 to 1.1 V applying pulse amplitude (ΔE) = 50 mV and scan rate (ѵ) = 30 mVs^−1^.

For both electrodes, the square wave and differential pulse voltammograms were recorded, the calibration graphs were constructed, and the regression equations were computed.

### Analysis of laboratory-prepared mixtures of BUM and its alkaline-induced degradant

Accurate aliquots of BUM and its alkaline-induced degradant were transferred separately from their stock solutions (1.0 × 10^–3^ M) into two series of 10-mL volumetric flasks for nRGO and CPE, respectively. The volume was completed with BR buffer (pH 3.0) to prepare different percentages of the degradant (1- 80%). The peak currents (Ip) at nRGO and CPE were measured at a scan rate of 100 mVS^−1^ using different pulse voltammetry. BUM concentrations were obtained from corresponding regression equations.

### Application to pharmaceutical formulation

The contents of ten Octomotol® tablets were finely ground, and mixed well, and the average weight of one tablet (110 mg of BUM) was accurately transferred into a 100 mL volumetric flask, dissolved in the least amount of methanol, and the volume was completed to 50 mL using distilled water. The flask contents were sonicated for 30 min until complete dissolution. The excipients were separated by filtration into a 100 mL volumetric flask and diluted to a final volume with distilled water. A working solution (0.8 mg/mL BUM) was prepared by taking a suitable aliquot from the stock solution and diluting it with distilled water. The square wave voltammograms were recorded following the outlined analytical voltammetric procedure (described under 2.6), and the nominal content was calculated using the corresponding regression equation. The standard addition method was applied by spiking the pharmaceutical formulation with different amounts of the standard drug substance, and the recoveries and RSD% were calculated.

### Spiked serum analysis

1.0 mL of ethanol and 0.5 mL of 5% ZnSO_4_ were added to 1.0 mL of serum, and the mixture was centrifuged for 15 min at 13,000 rpm. An aliquot (1 mL) of the clear solution was added to 14 mL BR buffer. The solution was de-aerated for 5 min and spiked with 150 ng mL^−1^ of BUM. The procedure was completed, as described above.

### Spiked urine analysis

A mixture of 1.0 mL urine, 1 mL 5% ZnSO_4,_ and 1.0 mL ethanol was added to 0.1 mL of 1 M NaOH to obtain pH 11. The mixture was centrifuged for 15 min at 13,000 rpm. An aliquot (1 mL) of the clear solution was added to 14 mL BR and spiked with 150 ng mL^−1^ of BUM. The procedure was completed, as described above.

### Chronoamperometry measurements

Chronoamperometric measurement of BUM at 10% nRGO electrode was studied by setting the electrode potential at + 850 mV; chronoamperograms were obtained by varying BUM concentrations from 3.3, 6.6, 10, 13.3, and 16.6 × 10^–5^ μmol L^−1^ in BR buffer (pH 3).

### EIS measurements

The parameters in an electrochemical impedance experiment were as follows: the oxidation potential value with an amplitude of 810 mV was studied at a frequency range of 0.1 Hz and 100 kHz. These parameters were applied on nRGO electrodes as well as CPE electrodes and tested in 10 mmol L^−1^ BUM.

## Results and discussion

Recently, the analysis of drugs by voltammetric methods in pharmaceutical and biological samples has risen rapidly, and their development and application for routine pharmaceutical analysis continue to be of interest. Moreover, with the progress in the field of nanotechnology, the synthesis of different electrode biosensor materials is making it more feasible for selective and low-limit detection of drugs. The electrochemical electrodes have the advantages of being of higher sensitivity and easier to prepare and operate. The use of the innovative material nRGO in the monolayer graphene is a promising candidate to detect the studied drug in accordance with charge transfer between the adsorbed molecules and graphene, which is characterized by high electrical conductivity, high carrier mobility, and a very high surface area^22^, recommending the use of nRGO for creating a sensitive biosensor for detection and quantification.

In this study, voltammetric-sensitive and selective nRGO and CPE electrodes were prepared and evaluated. A modified surface 10% nRGO electrode was more sensitive than those of the other electrodes due to its comparatively higher porosity and higher electro-active surface area, leading to a greater response of peak current towards the drug with the advantages of high selectivity and sensitivity and lower detection limits. DPV was applied for the determination of BUM in bulk powder, tablet, in the presence of its alkaline-induced degradant, and biological fluid samples. Chronoamperometry and EIS were also studied.

### Characterization of nRGO and CPE Electrodes

The active surface area of nRGO and CPE electrodes at different serial scan rates was determined by the application of the Randles–Sevcik equation^21^ for a reversible reaction using the universal probe 1.0 × 10^–3^ M K_3_Fe(CN)_6_ in 0.1 M KCl.

$${\text{I}}_{{{\text{pa}}}} = \, \left( {{2}.{69} \times {1}0^{{5}} } \right)\;{\text{A}}\;{\text{n}}^{{{3}/{2}}} {\text{D}}_{{\text{R}}}^{{{1}/{2}}} {\text{C}}_{0} v^{{{1}/{2}}}$$

where **I**_**pa**_ refers to the anodic peak current, **D**_**R**_ is the diffusion coefficient, ${\varvec{v}}$ is the scan rate, n is the number of electrons transferred,** C**_**0**_ is the concentration of K_3_Fe(CN)_6_, and **A** is the surface area of the electrode.

Cyclic voltammetry was applied using 1.0 × 10^–3^ M K_3_Fe(CN)_6_ in 0.1 M KCl electrolyte: n = 1, D_R_ = 7.6 × 10^−6^cm^2^s^−1^. The active surface area of the studied electrodes was then calculated from the slope of the plot of I_pa_ versus $v$^½^^23^. It was found to be 0.40, 0.15, 0.09, 0.07, and 0.025 cm^2^ for surface 10% nRGO (modified), 20% nRGO, 15% nRGO, 5% nRGO, and CPE electrodes, respectively.

The electroactive surface area of the 10% nRGO electrode was higher than that of other studied electrodes, resulting in a greater response of peak current towards BUM. Thus, the 10% nRGO electrode has the advantages of being of higher selectivity, sensitivity, and lower detection limits. Similar results were obtained for graphite pencil electrode in the determination of two non-classical b-lactams^20^.

### Voltammetric behavior of BUM at nRGO and CPE electrodes

Cyclic and differential pulse voltammetric techniques were applied to investigate the electrochemical process occurring on the surface of both nRGO and CPE electrodes^20^.

**Figure 1:**
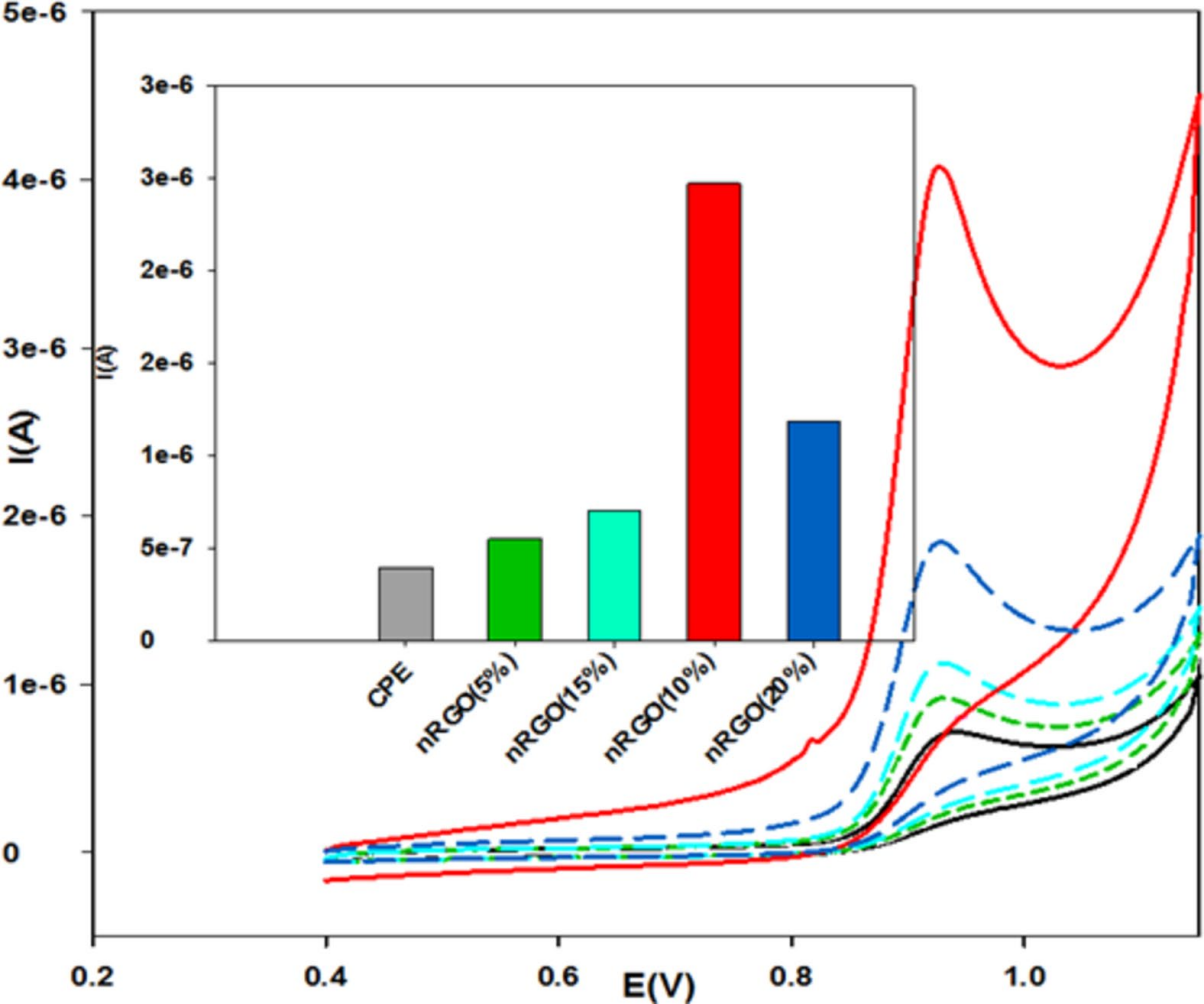
Comparative cyclic voltammograms of BUM employing nRGO and CPE electrodes at pH 3.0 using 0.04 M BR buffer, scan rate = 0.1 V s^−1^; [BUM] = 0.1 mM (vs. Ref. electrode Ag**|**AgCl**|**KCl 3 M).

The voltammetric behavior of BUM was investigated using different concentrated electrodes of nRGO (5, 15, and 20%), surface 10% nRGO, and CPE electrodes. BUM showed a well-defined single anodic peak with all electrochemical techniques. Cyclic voltammograms of 0.1 mM of BUM recorded in BR buffer solution (pH 3.0) at scan rates of 0.1 Vs^−1^ exhibited a well-defined single anodic peak at about 0.92 for all electrodes (Fig. 1). The current intensity of the oxidation peaks obtained from the surface 10% nRGO electrode was much higher as compared to the peaks at nRGO electrode at (5, 15, and 20%) concentrations and peak at CPE electrode, indicating that the electro-active surface of 10% nRGO is greater than those of the other electrodes, as shown in Fig. 1.

**Figure 2:**
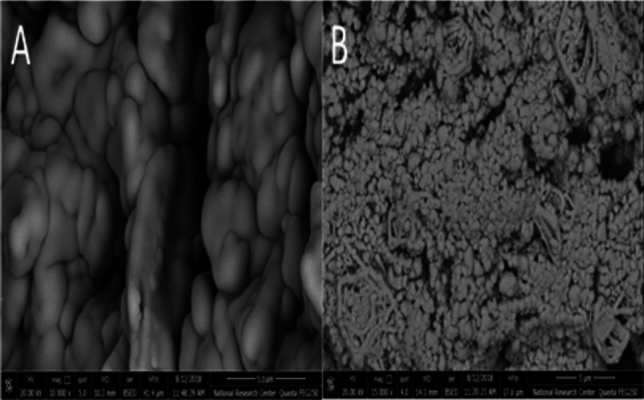
SEM micrographs of CPE (**A**) and 10% nRGO (**B**) electrodes.

This remarkable enhancement in the 10% nRGO peak current can be attributed to the comparatively higher porosity of nRGO^10,20,24^ as displayed by the scanning electron microscope, shown in Fig. 2, thus supporting its higher sensitivity and lower detection limits. Subsequently, 10% nRGO was selected as the electrode of choice for application and further investigation throughout this study.

These anodic peaks may be attributed to the oxidation of the secondary amine^6,25^. However, no corresponding cathodic peaks were observed on the reverse scan, signifying the irreversibility of the electrodes process.

The differential pulse voltammograms of the same concentration of the BUM and under the same conditions showed a single peak at 0.7 V using a surface 10% nRGO electrode, while its alkaline degradant did not show such a peak.

**Figure 3:**
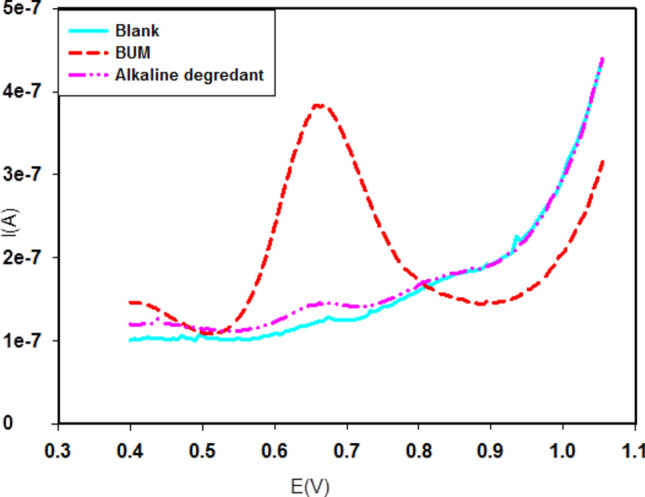
Differential pulse voltammograms of BUM and its alkaline degradant against a blank of supporting electrolyte of 0.04 M BR buffer (pH 3.0), pulse amplitude = 50 mV, pulse time = 0.04 s, scan rate ($v$) = 30 mV s^−1^, using 10% nRGO electrode; [BUM] = 0.1 mM (vs. Ref. electrode Ag***|***AgCl**|**KCl 3 M).

Meanwhile, the developed DPV technique facilitated the determination of the drug BUM in the presence of its alkaline degradant. As such, the proposed method is considered a stability-indicating one. This is attributed to the disappearance of the alkaline degradant wave at the SW voltammograms of BUM, as shown in Fig. 3.

### Optimization of experimental conditions

#### Effect of supporting electrolyte

The universal buffer (BR) was used in the study as the best signal was produced. Weak peaks appeared when other buffers (acetate, borate, citrate, and phosphate) were used.

#### Effect of pH

**Figure 4:**
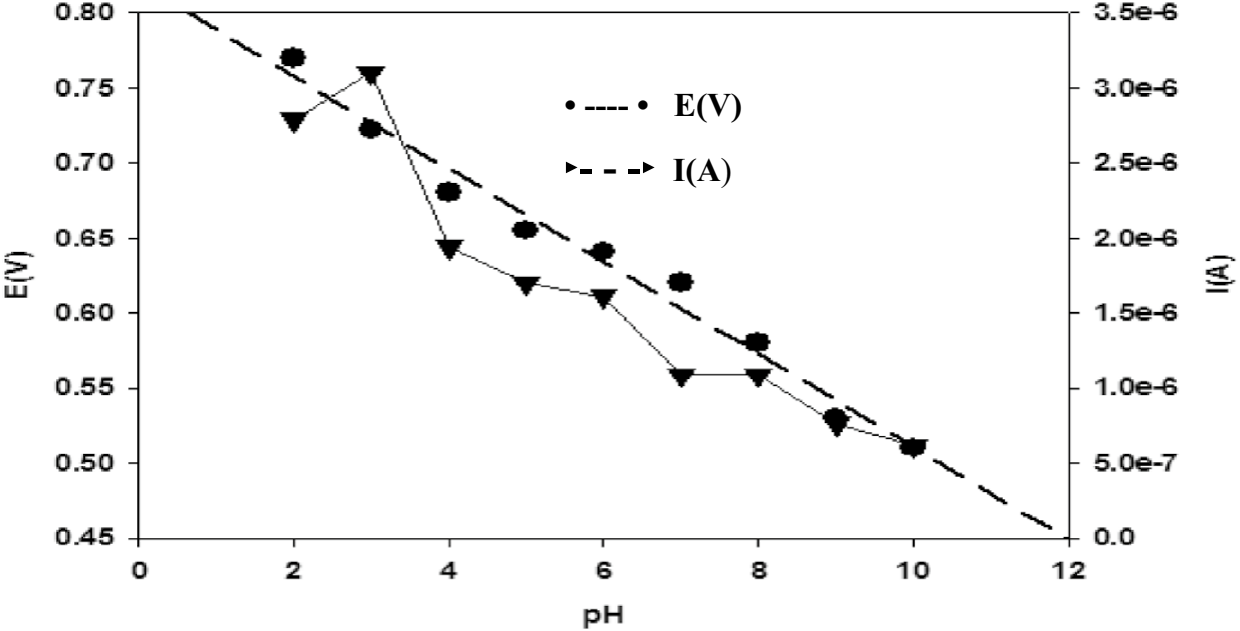
Effect of pH on peak current and the relation between different pH and potential at nRGO electrode; $v$ = 40 mVs^−1^, ΔE = 50 mV, and pulse time = 0.04 s using DPV (vs. Ref. electrode Ag|AgCl|KCl 3 M).

Experiments at different pH values (2.0–12.0) were carried out to assess the pH impact on the monitored electro-analysis signal at the nRGO electrode. The observed signals were pH-dependent since sharp electrochemical currents were only detected in acidic media. SWV studies showed that the anodic peak current was nearly constant. From pH 2.8 to 3 at the nRGO electrode and after pH 3, the peak height fluctuated, decreasing till pH 12.0, after which no distinct peak was noticeable, as shown in Fig. 4. Therefore, pH 3.0 was used in all further measurements. It also shows the dependence of the peak potential on pH. The oxidation peak potential of BUM moves towards negative values with the increase in pH of the medium, signifying that protons are involved in the electrode reaction process. A linear correlation between pH and anodic peak potential E(V) was found in the pH range of 2.0–12.0 (Fig. 4), with a linear equation and regression coefficient as follows:

$${\text{E}}\left( {\text{V}} \right) = 0.{8193} - 0.0{3}0{\text{9 pH}}\quad \left( {{\text{R}}^{2} = 0.{982}} \right)$$

#### Effect of scan rate

**Figure 5:**
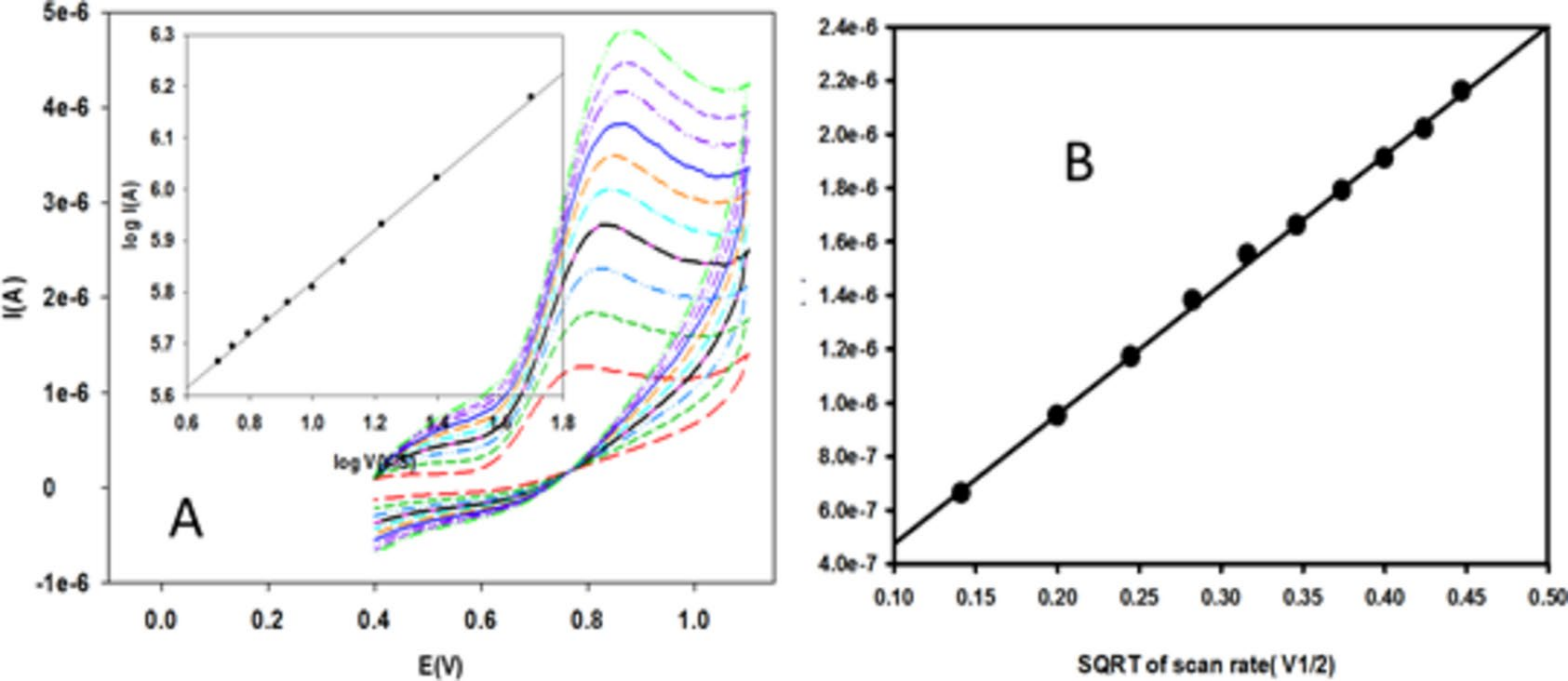
Relation between current and scan rate for oxidation of BUM (vs. Ref. electrode Ag|AgCl|KCl 3 M).

Studying the behavior of BUM at 10% nRGO electrode surface by measuring the scan rate ($v$) versus the peak current (I) indicates that the process is a diffusion-controlled mechanism^23^. This behavior was assessed by applying the Randles–Sevcik plot (Ip vs. *v*^1/2^^20^ on measuring the scan rate from 20 to 500 mVs^−1^, where a linear relationship was obtained when the current (I) was plotted against $v$^1/2^ alongside the scanning rate (R^2^ > 0.99), indicating mass transport exact diffusion, as illustrated in Fig. 5.

In addition, a plot of log (I) against log ($v$) of the BUM solution gave a straight line with a slope value of 0.508, which is close to 0.5, evidence of a diffusion-controlled mechanism^26^ for the submitted electrode. The high sensitivity as well as the well-shaped voltammetric curves were found at 80 mVs^−1^.

**The BUM oxidation mechanism**

**Figure 6:**
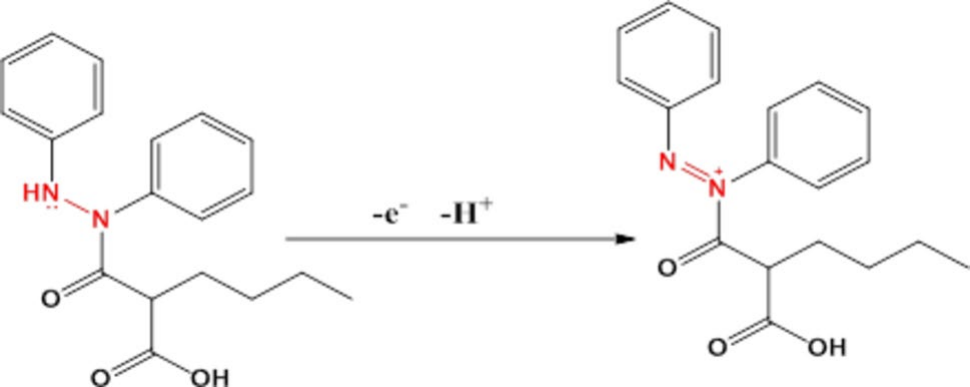
Proposed oxidation mechanism of BUM at nRGO and CPE.

For a completely irreversible electrode process, the oxidation peak potential and scan rate are described by the Laviron equation^27,28^:

$$E_{{\overline{\overline{p}} }} E^{0} - \left( {\frac{RT}{{\alpha nF}}} \right)In\left( {\frac{{TRk^{0} }}{\alpha nF}} \right) + \left( {\frac{2.303RT}{{\alpha nF}}} \right)In v$$

where $E_{{\overline{\overline{p}}}}$ stands for oxidation peak potential and E^0^ stands for the formal potential, α is the transfer coefficient, n is the electron transfer number, T is the temperature (298 K), k^0^ the standard heterogeneous rate constant of the reaction (s^−1^), $v$ represents the scan rate, R is the universal gas constant (8.314 Jmol^−1 ^K^−1^) and F is the Faraday constant (96.480 Cmol^−1^). For an irreversible reaction, consider α = 0.5; hence, for peak signal, the slope was equal to 0.0348 at nRGO, indicating the transfer of one electron. Accordingly, the expected mechanism for the reaction at the electrodes’ surface is proposed, as shown in Fig. 6. BUM has one electroactive site, namely the secondary amine, which is characterized by its high electron density, where oxidation is most likely to occur.

#### Effect of surfactant

**Figure 7:**
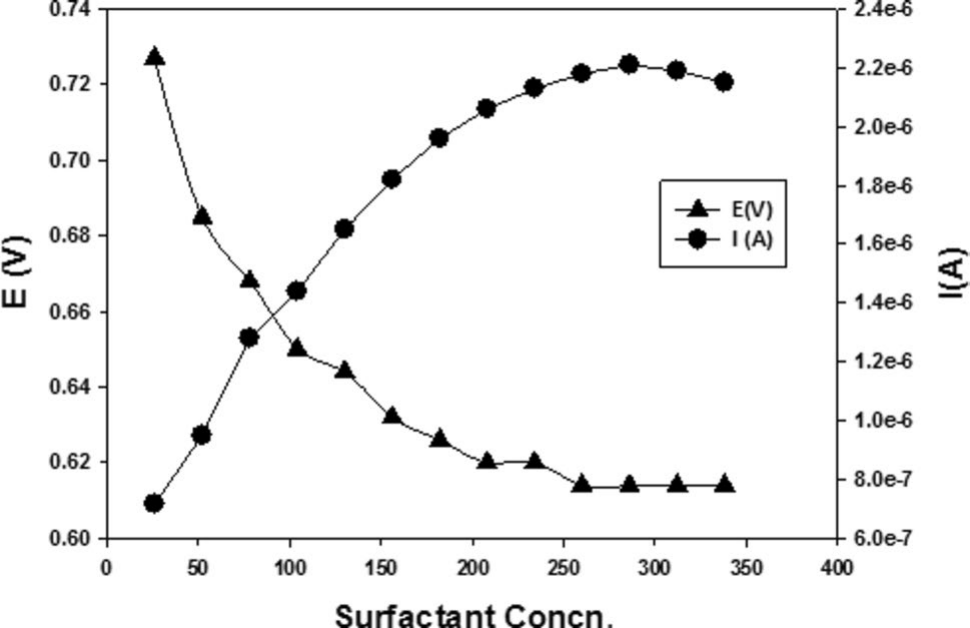
The SWV response of BUM in BR buffer, pH 3.0 on nRGO upon additions of SDS at different concentrations (vs. Ref. electrode Ag**|**AgCl**|**KCl 3 M).

SWV response of 0.1 mM of BUM in BR buffer, pH 3.0, on nRGO upon successive additions of sodium dodecyl sulfate (SDS) surfactant is shown in Fig. 7. Increasing surfactant concentration resulted in a current increase with decreased potential. The increase in current in the presence of the surfactant could be attributed to the aggregation of surfactant molecules on the electrode surface in the form of a bilayer when the electroactive species approach the electrode surface. The electron transfer can take place by two main probabilities to allow charge transfer: displacement of the adsorbed surfactant by the analyte BUM and/or reaching the electrode surface through the space of one to two head groups of surfactant moieties. It was concluded that upon the addition of different concentrations of SDS surfactant, the current increased with decreasing the potential and approached zero as a measure of increasing the sensitivity and removing any interferences with cations.

## Chronoamperometry measurements

Chronoamperometry is used to study the diffusion process of the working 10% nRGO electrode, where the potential step is applied to the electrode, and the resulting current is monitored as a function of time. Before beginning the experiment, the electrode was held at a potential at which no faradaic process occurred; then the potential was stepped to a value at which a redox reaction occurred. Zero time is defined as the time at which the potential step is initiated.

**Figure 8:**
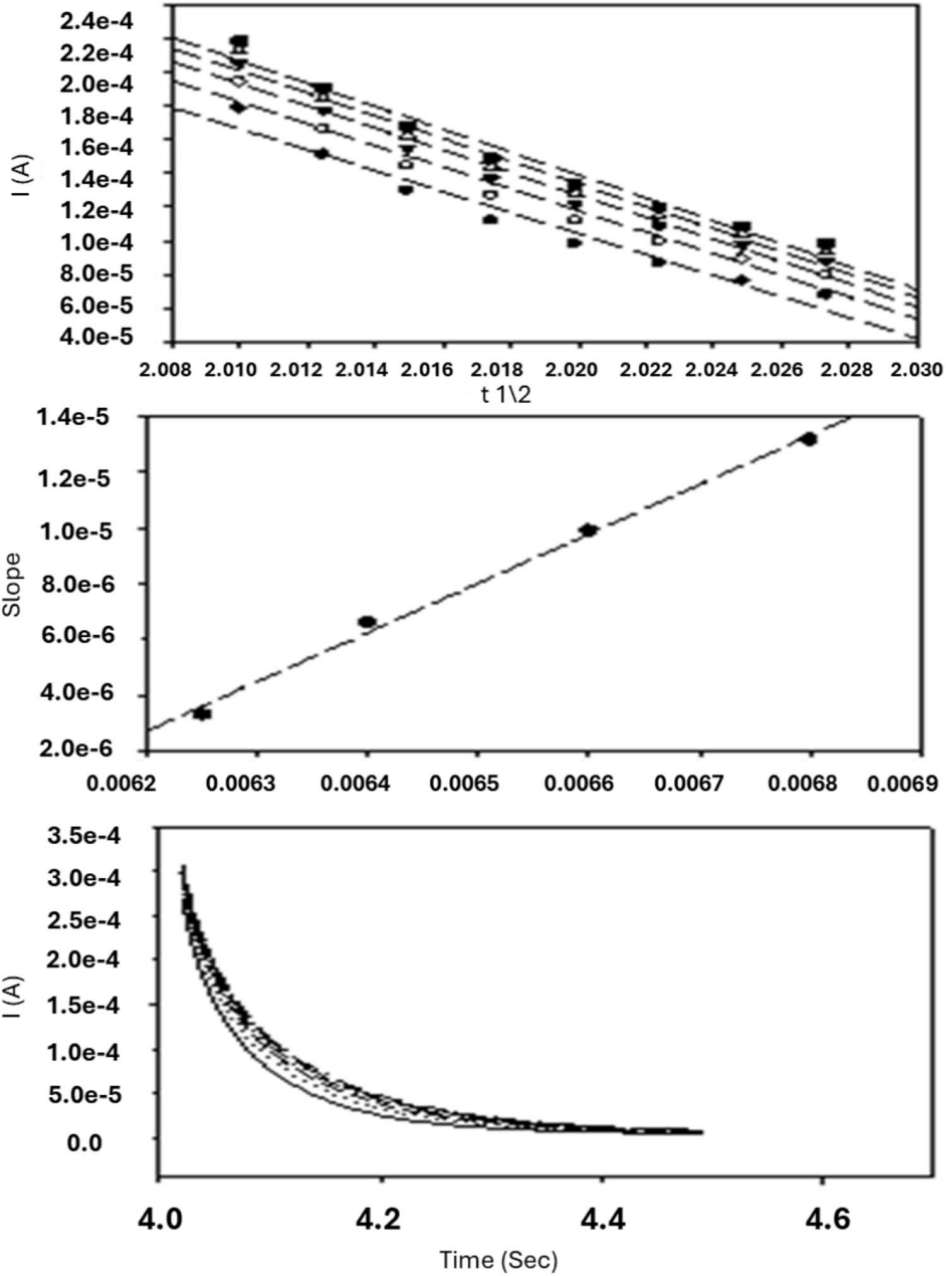
Chronoamperometric measurements of BUM electro-oxidation obtained with a 10% nRGO electrode in different BUM concentrations.

Chronoamperometric measurements of BUM at a 10% nRGO electrode generate high charging currents, which decay exponentially with time, as described in the Cottrell equation^29^, as shown in Fig. 8.

Experimental plots of I versus t^1/2^ were employed. The slopes of the resulting straight lines were then plotted versus BUM concentration. D was calculated from the resulting slope and Cottrell equation; the mean value was found to be 8.15 × 10^–5^ cm^2^ S^−1^.

## EIS studies

Studying the interface characters of the surface nRGO and CPE electrodes was carried out by EIS^19^. The EIS data was obtained for the studied electrodes at frequencies varying between 0.1 Hz and 100 kHz with an applied potential in the region corresponding to the electrolytic oxidation of BUM in BR buffer pH 3.

**Figure 9:**
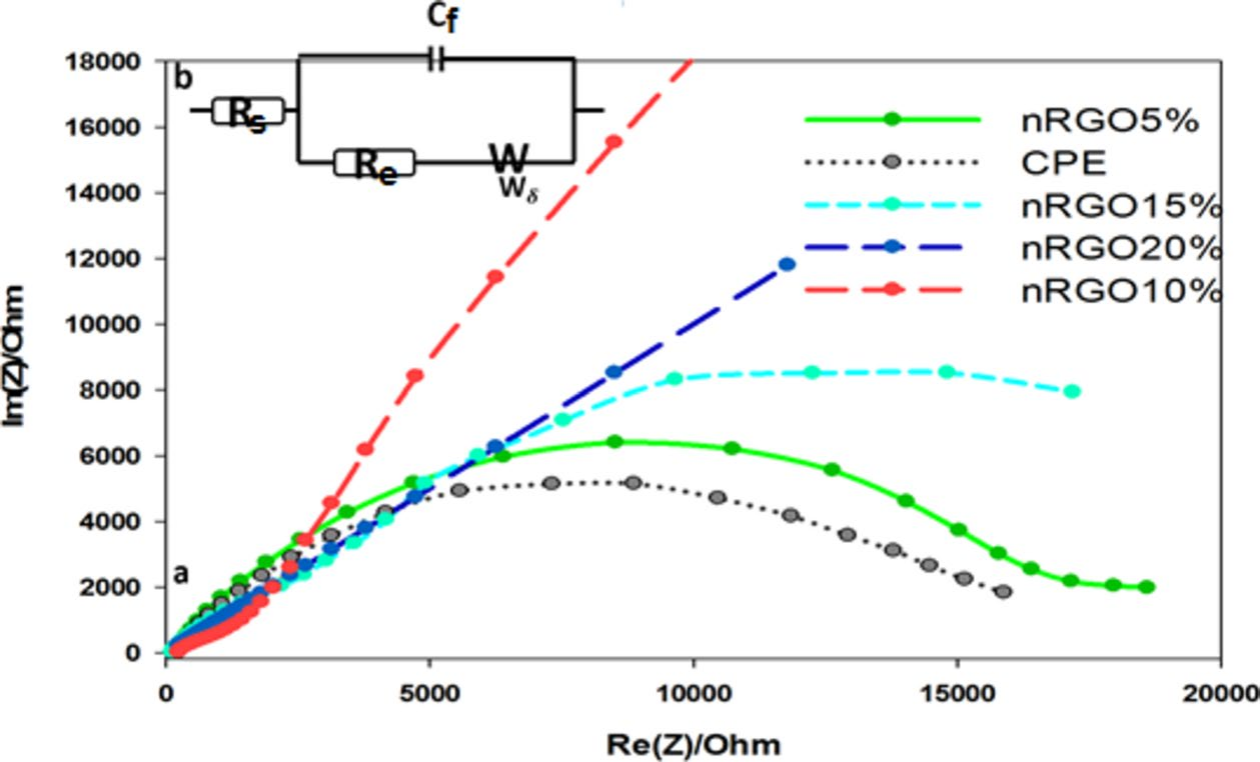
Nyquist plots of impedance spectra of BUM using 5, 10, 15, 20% nRGO, and CPE at the oxidation potential of 810 mV (b) equivalent circuit model used in the fit procedure of the impedance spectra.

Figure 9 shows a typical impedance spectrum presented in the form of a Nyquist plot of BUM using 5, 10, 15, 20% nRGO, and CP electrodes at the oxidation potential of 810 mV. The impedance responses of BUM displayed a great difference between the five studied electrodes; in the case of CPE, the impedance spectra of BUM show a semicircle with a larger diameter, which corresponds to high frequency with a little improvement through the 5, 15, and 20% nRGO electrodes. In the case of a 10% nRGO electrode, the diameter of the semicircle decreases distinctly, the charge transfer resistance of electrooxidation of BUM declines significantly, and the charge transfer rate is improved (Fig. 9).

A simple equivalent circuit model (Fig. 9) was used to obtain the data that illustrates full information for the impedance spectroscopy. The resulting experimental data were compared to an “equivalent circuit”, where **R**_**s**_ is the solution resistance, **R**_**e**_ is the electrode resistance, **C**_**f**_ is the capacitance of the double layer, and **W** is the Warburg impedance due to diffusion. The electronic charge transfer resistance (**R**_**e**_) showed a clear decrease in values in the case of the 10% nRGO electrode compared to the others, indicating less interfacial electronic transfer resistance, more facilitation of charge transfer, and a higher capacitative component value. This could be the result of a selective interaction between the drug and the 10% nRGO electrode, resulting in an increase in the current signal of the electro-oxidation process.

**Figure 10:**
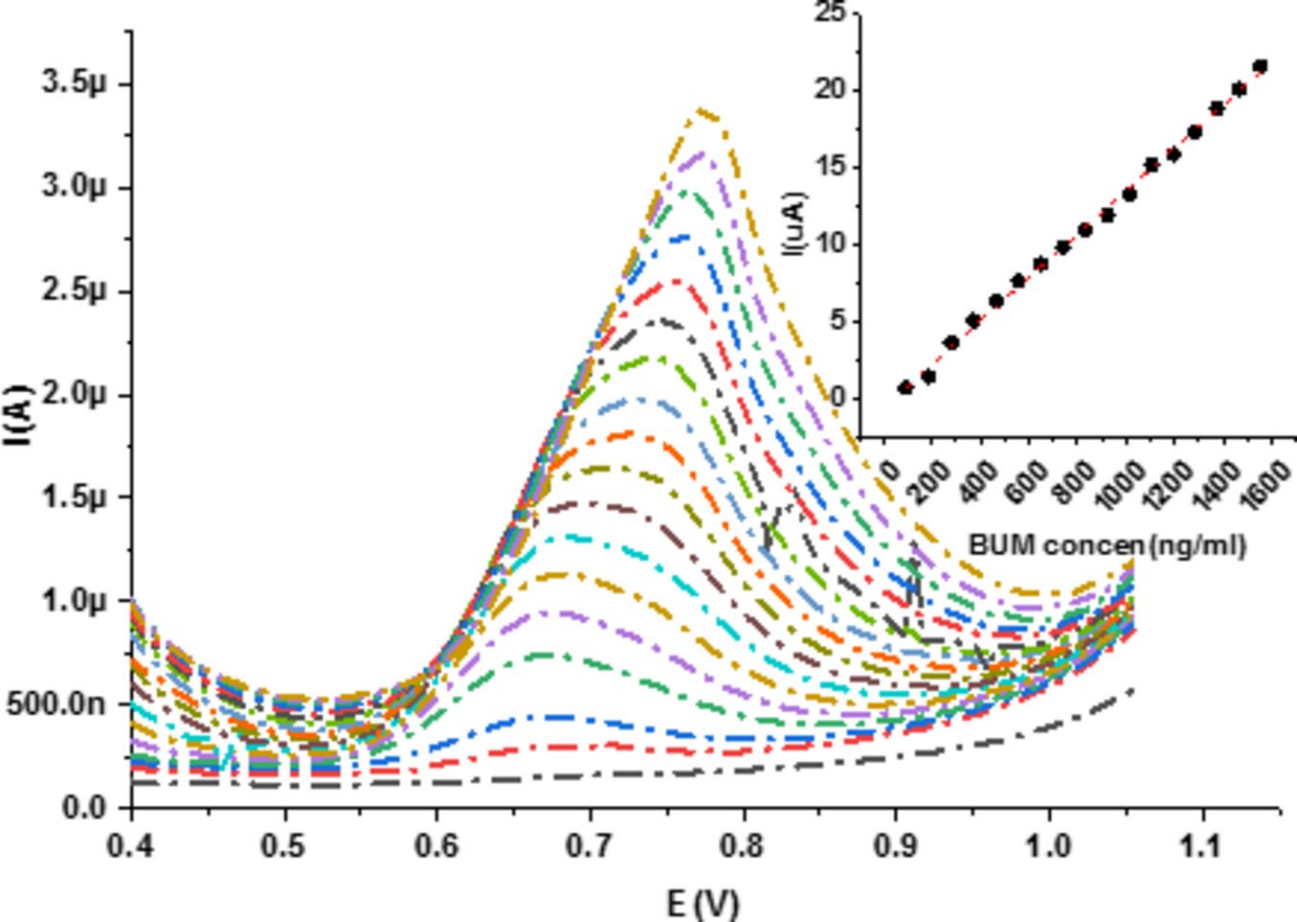
Square wave voltammograms relating the peak current intensity to different concentrations of BUM at nRGO in 0.04 M BR buffer solution (pH 3.0), preconcentration time = 10 s, pulse amplitude (DE) = 50 mV, pulse time = 0.04 s, and scan rate ($v$) = 130 mV s^−1^ (vs. Ref. electrode Ag|AgCl|KCl 3 M).

The square wave voltammograms relating the peak current intensity to different concentrations of BUM at nRGO in 0.04 M BR buffer solution (pH 3.0) are shown in Fig. 10, and the best fitting values calculated from the equivalent circuit for the impedance data at 810 mV are listed in Table 1.

**Table 1:** Electrochemical impedance spectroscopy fitting data corresponding to Fig. 10.

Parameter	10% nRGO	20% RGO	15% nRGO	5% nRGO	CPE
Rs	208.6	215.7	319.8	445.8	316
Re	0.0000113	54.61	47.1	0.5399	14.45
Cf	0.09173 × 10^–6^	8.162 × 10^–6^	0.7247 × 10^–6^	0.1804 × 10^–6^	0.05469 × 10^–6^
S	649 441	42 310	100 839	172 922	97 395

R_s_, solution resistance, R_e_, is the electrode resistance, C_f_, capacitance of the double layer, S, Warburg diffusion.

From Table 1, it is obvious that the electronic charge transfer resistance (Re) shows a noticeable decrease in values in the case of 10% nRGO electrode compared to 20% nRGO, 15% nRGO, 5% nRGO, and CP electrodes, which indicates less electronic resistance and more facilitation of charge transfer of 10% nRGO > 20% nRGO > 15% nRGO > 5% nRGO > CP electrodes. The capacitive component value of 10% nRGO is relatively higher compared to those of the studied electrodes. This is attributed to the increase in ionic adsorption at the electrode/electrolyte interface. Also, the decrease in the interfacial electron transfer resistance due to the selective interaction between 10% nRGO and BUM resulted in the observed increase in the current signal for the electro-oxidation process.

## Method validation

The validity of developed methods was evaluated according to the International Council for Harmonization (ICH) guidelines on validation of analytical procedures (Q2R1)^30^. They were validated by evaluating linearity, range, precision, accuracy, limit of detection (LOD), limit of quantitation (LOQ), specificity, and robustness.

### Linearity and range

In the developed technique, quantification of BUM at 10% nRGO was based on the dependence of the peak current (I_p_) on the drug concentration in the analyzed BR solution after optimizing the above experimental parameters. Linearity was achieved over the concentration range of 0.9 × 10^2^–15 × 10^2^ ng mL^−1^ for BUM, and the corresponding regression equation was computed and presented in Table 2.

**Table 2:** Regression parameters obtained from SWV analytical curves of BUM at 10% nRGO.

Parameter	10% nRGO
a (µA cm^−2^)	− 0.466
b (µA cm^−2^ mLng^−1^)	0.014
_Sa_ (µA cm^−2^)	0.113
S_b_ (µA cm^−2^)	0.00018
S_y/x_ (µA cm^−2^ mLng^−1^)	0.33762
LOD (ng mL^−1^)	26.63
LOQ (ng mL^−1^)	80.71
R	0.99872
r^2^	0.99745
LR (ng mL^−1^)	0.9 × 10^2^–15 × 10^2^
N	17

A, intercept; b, slope; s_a_, standard deviation of intercept; S_b_, standard deviation of slope; S_y/x_, error standard deviation; LOD, limit of detection; LOQ, limit of quantification; R, correlation coefficient; r^2^, coefficient of determination; LR, linear range; and N, number of data.

### Limit of detection (LOD) and limit of quantitation (LOQ)

LOD and LOQ values of BUM were calculated for 10% nRGO using the following equations: LOD = 3.3 $\sigma /S$ and LOQ = 10 $\sigma /S$ (where $\sigma$ is the standard deviation of the peak current in µA and S is the slope of the calibration graph). The results obtained are presented in Table 2.

### Accuracy and precision

The accuracy of the proposed method was determined as a percentage recovery of different concentrations within the linearity range.

*Repeatability and Intermediate Precision*

The intra- and inter-day precision of the method was evaluated by assaying freshly prepared solutions in triplicate on the same day and on three different days, respectively. The repeatability (intra-day) and reproducibility (inter-day) results obtained by means of the proposed SWV procedure are presented in Table 3. The % RSD values of intra- and inter-day studies were less than 2%, indicating that the developed method was precise.

**Table 3:** Inter- and Intra-day precision of the voltammetric determination of BUM.

Concentration (ng mL^−1^)	10% nRGO electrode		CPE	
	Intra-day M ± % RSD*	Inter-day M ± % RSD*	Intra-day M ± % RSD*	Inter-day M ± % RSD*
200	99.97 ± 0.15	100.05 ± 0.081	99.6 ± 1.45	101.01 ± 1.27
500	100.03 ± 0.11	100.08 ± 0.06	101.4 ± 0.65	99.21 ± 1.01
800	100.02 ± 0.058	99.97 ± 0.102	100.8 ± 1.89	99.50 ± 0.78
M ± %RSD**	100.01 ± 0.035	100.03 ± 0.056	100.60 ± 0.916	99.90 ± 0.966

*Each result is the average of three separate determinations.

**Average of nine determinations.

The accuracy of the proposed SWV method was assessed by applying the standard addition technique; the results obtained are presented in Table 4.

**Table 4:** Determination of BUM in tablet formulation using standard addition technique.

Pharmaceutical (ng mL^−1^)	Found* % ± SD	BUM Pure added (ng mL^−1^)	Recovery %** of added BUM
400	98.92 ± 1.523	200	99.80
		400	99.01
200		600	101.01
		800	100.48
Mean ± RSD %			100.07 ± 0.865

*Mean of three determinations.

**Average of three replicates experiments.

### Specificity

The specificity of the proposed voltammetric method was proven by its ability to determine BUM in the presence of its alkaline degradant as well as in pharmaceutical preparation without interference from excipients and preservatives that are commonly present. This was confirmed by performing the method on a placebo sample. The high specificity obtained by the developed method for BUM at a 10% nRGO surface electrode was attributable to the mechanism of oxidation of the drug in relation to the pathway of the alkaline degradation, as shown in Table 5.

**Table 5:** Determination of BUM in the presence of alkaline degradant by the proposed SW voltammetric method.

Intact drug (ng mL^−1^)	Degradant%	Recovery %* 10% nRGO Sensor
200	80	100.06
400	40	99.99
600	20	100.05
800	10	100.03
Mean ± SD		100.03 ± 0.031

*Mean of three determinations.

### Robustness

The robustness of the proposed method was demonstrated by the constancy of the peak current with deliberate minor changes in the experimental parameters. The studied variables included the change in pH (± 0.2) and the time considered before each measurement (10 s ± 5 s). These minor changes that may have taken place during the experimental operation did not affect the peak current intensity of the studied drug, indicating the reliability of the proposed method during normal usage.

### Statistical analysis

The results of the assay of BUM by the proposed SWV method were compared with those obtained using the reference published HPLC method^6^ using Student’s t-test and variance ratio F-test^31^; it revealed that there is no significant difference between the two methods, as shown in Table 6. The calculated “t” and “F” values were lower than the theoretical values.

**Table 6:** Statistical comparison between the results obtained by the proposed method at 10% nRGO electrode and reference method.

Intact drug (ng mL^−1^)	10% nRGO	Ref. method^6^*
Mean ± SD	100.02 ± 0.054	100.04 ± 0.053
N	5	5
Sample variance	0.003	0.003
t-test (t-Tabulated = 2.31)**	0.590	–
F-test (F-Tabulated = 6.39)**	0.96	–

*Ref. method is HPLC.

**t-test and F-test between brackets are the theoretical values at *P* = 0.05.

### Application to Pharmaceutical tablets and biological fluids

The proposed method was applied to the quantitative analysis of BUM in pharmaceutical preparation and biological fluids. The results and recoveries of known amounts of BUM added to pharmaceutical preparation are given in Table 4, and for spiked serum and spiked urine in Tables 7 and 8, respectively. The recovery ranged from 99.80 to 101.01% by standard addition method (Table 4). These results indicate that the proposed method is appropriate for the quality control of the drug in its tablet dosage form. No oxidation compounds and no extra noise peaks present in different matrices occurred in the potential range where the analytical peak appeared.

**Table 7:** Application of the proposed method for determination of BUM in spiked serum samples at 10% nRGO surface electrode**.**

Added (ng mL^−1^)	Found (ng mL^−1^) (Mean ± SE*)	% Recovery	% RSD**
100	99.92 ± 0.031	99.92	0.031
250	250.10 ± 0.104	100.04	0.103
300	300.11 ± 0.130	100.04	0.130
500	499.85 ± 0.113	99.97	0.113

*SE: Standard error of five replicates.

** RSD: Relative standard deviation, an average of five replicate determinations.

**Table 8:** Analytical performance data of spiked urine at 10% nRGO surface electrode.

	SWV		
Added (ng mL^−1^)	150.0	300.0	500.0
Found*	150.07	299.93	500.15
recovery	100.047	99.977	100.03
Bias (%)	0.43	0.21	0.82
SD	0.062	0.121	0.041
SE	0.028	0.037	0.115
CL(P = 0.05)	150 ± 0.082	300 ± 0.110	500 ± 0.071
CV	1.120	0.760	0.421

SD, standard deviation; SE, standard error; CL, confidence level; and CV, coefficient of variation.

*Mean of five measurements.

The accuracy of the voltammetric method was determined by its recovery in spiked experiments. The results in Tables 7 and 8 demonstrate that the SWV method has adequate precision and accuracy and, consequently, can be applied to the determination of BUM in pharmaceuticals and biological fluids without any interference.

## Greenness and whiteness evaluation

### Greenness evaluation

Green analytical methods are important as they reduce energy input, solvent use, waste production, and operator exposure, and avoid the use of toxic organic solvents.

In this study, the Eco-Scale (ES) grading system and Analytical Greenness Calculator (AGREE) were used to evaluate the greenness of the developed analytical voltammetric method for the simultaneous analysis of the BUM in the presence of its alkaline degradant as well as in spiked serum and urine samples and tablets. Both are well-established methods reported to provide reliable and precise results about method greenness^32^.

The results obtained (Table 9) illustrate the superiority of the proposed technique from a greenness standpoint.

**Table 9:** Greenness profiles of the proposed method and reported method using ES and AGREE tools.

Eco-Scale:		
Parameter	Penalty points	
	Proposed method	Reported method^6^
(i) Reagents:		
Ethanol	4	–
*Britton-Robinson buffer*	0	–
Acetonitrile	-	8
Methanol	6	12
*Sodium hydroxide*	2	–
*Dimethylformamide*	5	–
*SDS*	0	–
*Graphite*	0	–
*nRGO*	0	–
*Paraffin oil*	2	–
(ii) Instruments		
Energy consumption	0	1
Occupational hazard	0	0
Waste	0	5
Total penalty points	19	26
Total Score	81	74
Greeness	Excellent for analysis	Good for analysis

AGREE tool:		
Parameter	Proposed method	Reported method
Pictogram indicating the final score		
Score	0.83	0.65
Greeness	Excellent green	Acceptable green

For the AGREE metric, a 12-segment circular pictogram was produced using AGREE open-source software. It addresses all 12 aspects of green analytical chemistry (GAC). Each segment’s color ranges from deep green to deep red depending on how the analytical technique affects the environment, with an overall evaluation score estimated in the pictogram’s center; as shown in AGREE pictograms (Table 9).

Another green assessment, the ES method, was applied to compare the proposed and reported^6^ methods with overall evaluation scores, confirming the greenness superiority of the proposed technique (Table 9).

### Whiteness evaluation

**Figure 11:**
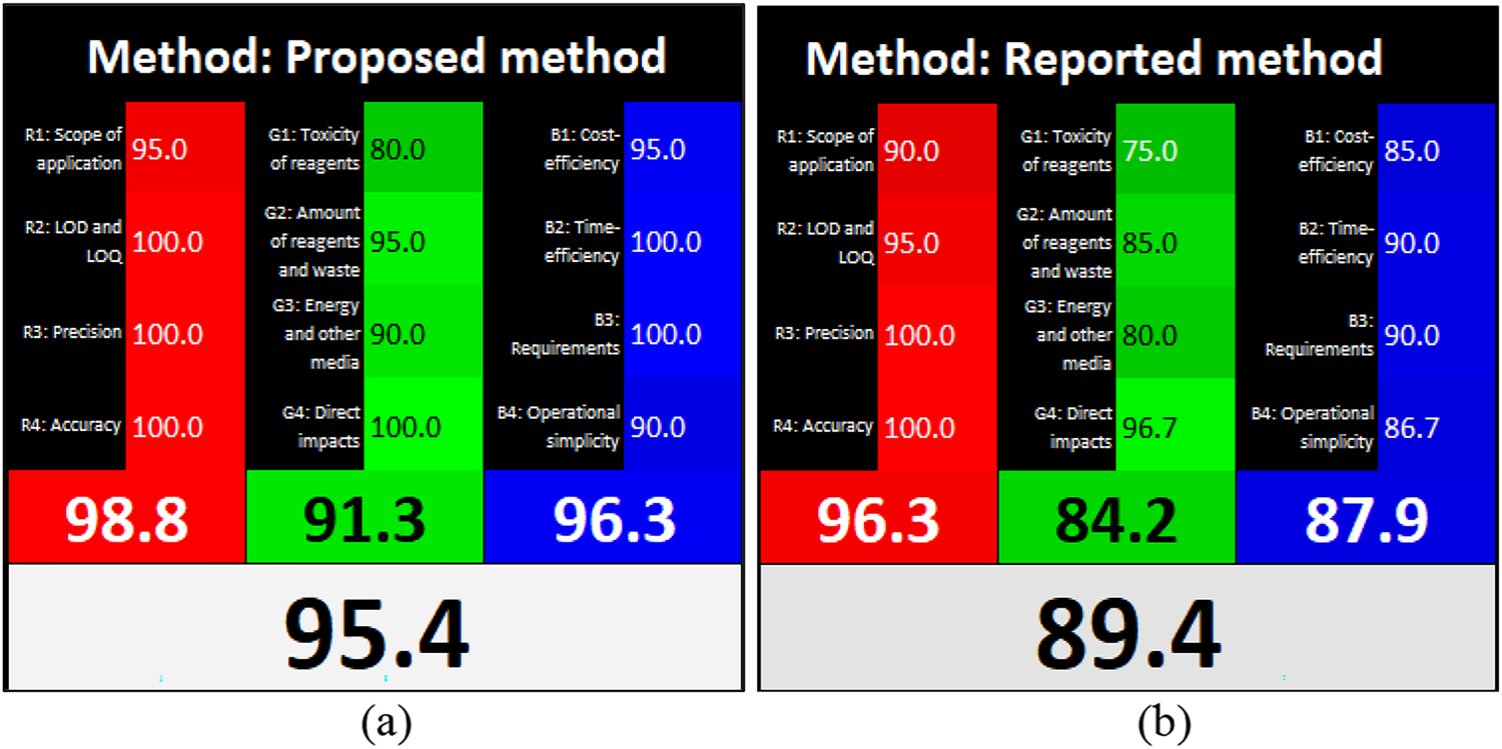
Whiteness evaluation of the proposed and reported methods by the RGB algorithm.

The RGB12 model was utilized to assess the proposed technique’s whiteness per the 12 White Analytical Chemistry guiding rules^33,34^. This model is made up of 12 Excel-primed algorithms categorized into red, green, and blue color groups. According to the validation criteria for sensitivity (LOD and LOQ), precision, and accuracy, the analytical effectiveness is assessed in the red category. On the other hand, the GAC rules fall under the green category. The blue category assesses the technique’s performance using factors like operational ease, time effectiveness, minimum practical needs, and cost-effectiveness. The whiteness ratings for both proposed and reported^6^ techniques are depicted in Fig. 11, confirming the superiority of the proposed technique regarding whiteness.

## Developed method’s practicality (Blueness evaluation)

**Figure 12:**
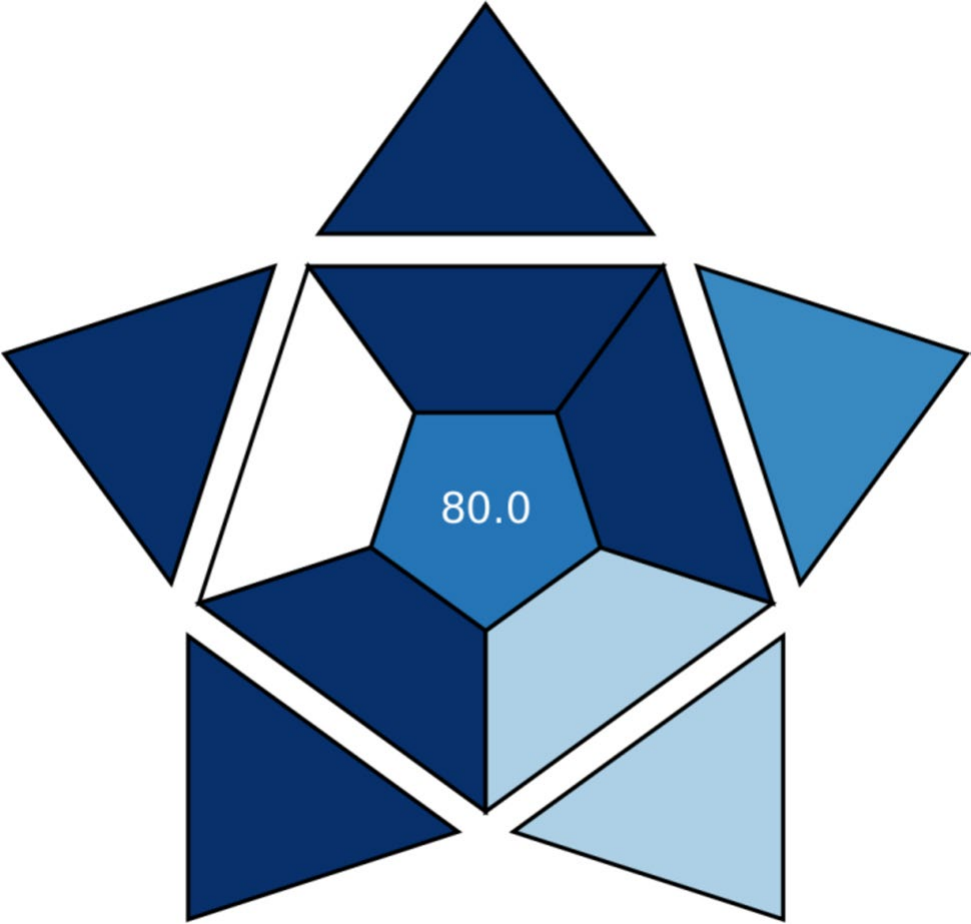
Blueness assessment of the developed voltammetric method by the BAGI tool.

To evaluate the developed method’s practicality, a new metric tool named the Blue Applicability Grade Index (BAGI) is utilized^35^. The BAGI tool generates two types of results: an asteroid pictogram (as a graphical representation) and a numerical rating score. The pictogram visually represents the evaluation findings using multiple hues of blue, where dark blue denotes high compliance, blue represents moderate compliance, light blue indicates low compliance, and white signifies non-compliance. Both the pictogram and the rating score, which are based on ten criteria, demonstrate the practicality and/or functionality of the developed method^35^. For the method to be considered “practical,” the final rating score should exceed 60. Figure 12 shows that the developed voltammetric method received a high rating score of 80.

## Conclusion

A highly sensitive, precise, selective, and convenient cyclic voltammetric technique for the determination of BUM using nano-reduced graphene oxide electrode (10% nRGO) was developed and successfully applied for the simultaneous determination of BUM in pure forms, pharmaceutical formulation, and biological fluids with no possible interferences from alkaline degradants or matrices in pharmaceutical formulation or biological fluids. The results showed that the quantity of drug substance in drug products was in good agreement with the given labeled quantity. The proposed method can be used for routine quality control testing and as a stability-indicating method for the determination of the drug in the presence of its alkaline-degradant at nano concentrations. This method is considered the first nanoelectrochemical technique. It is highly safe and cost-effective, and serves as a superior eco-friendly alternative to other analytical methods, excelling in greenness and whiteness. Additionally, it demonstrates high practical effectiveness. It also allows for disposable electrode manufacture at low cost, and fast surface renewal and can be very useful in the construction of simple, sensitive disposable electrochemical sensors as a replacement for dull electrodes or complicated and expensive HPLC methods.

## Data Availability

All data and materials generated or analyzed during this study are included in this published article.
